# Intravenous Immunoglobulin in the Management of Lupus Erythematosus Panniculitis

**DOI:** 10.7759/cureus.6790

**Published:** 2020-01-27

**Authors:** Nada G AlQadri, Bayan AlNooh, Malak M AlTewerki, Ahmad Almotairi, Saad Alajlan

**Affiliations:** 1 Department of Dermatology, Alfaisal University College of Medicine, Riyadh, SAU; 2 Department of Dermatology, King Khalid University Hospital, Riyadh, SAU; 3 Department of Neuroscience, King Faisal Specialist Hospital and Research Centre, Riyadh, SAU; 4 Department of Pathology, King Saud University Medical City, Riyadh, SAU; 5 Department of Dermatology, King Faisal Specialist Hospital and Research Centre, Riyadh, SAU

**Keywords:** lupus, lupus erythematosus panniculitis, lupus erythematosus profundus, cutaneous lupus, sle, intravenous immunoglobulin, ivig, chronic cutaneous lupus

## Abstract

Lupus erythematosus panniculitis (LEP) is a rare variant of cutaneous lupus erythematosus (CLE). It is characterized by the presence of a chronic inflammatory process involving the deep dermis and subcutaneous tissues. It commonly presents as deep indurated nodules or sharply demarcated plaques. Antimalarial medications are considered first-line therapy for most cases of LEP while systemic corticosteroids are saved for more resistant lesions. Intravenous immunoglobulin (IVIG) is made up of concentrated polyclonal immunoglobulin G (IgG) fractionated from the blood of healthy blood donors. Nowadays, it is used for the treatment of numerous autoimmune and systemic inflammatory diseases. In this case, we report the case of a female with multiple LEP and discoid lupus erythematosus (DLE) lesions refractory to multiple standard therapy modalities that responded dramatically to IVIG.

## Introduction

Lupus erythematosus panniculitis (LEP), also known as lupus profundus (LP), is a rare subtype of lupus erythematosus (LE) consisting of 1%-3% of patients with cutaneous lupus erythematosus (CLE) [1]. LEP is a chronic inflammatory process that mainly involves the deep dermis and subcutaneous tissues, usually presenting as deep indurated nodules or sharply demarcated plaques [2]. It can either present as the sole manifestation of the disease or in association with discoid lupus erythematosus (DLE) or systemic lupus erythematosus (SLE). The most commonly involved areas include the lateral aspects of the arms and shoulders, buttocks, trunk, breast, face, and scalp [3]. Antimalarial medications are considered first-line therapy for most cases of LEP; meanwhile, systemic corticosteroids are saved for resistant lesions [2,4]. Intravenous immunoglobulin (IVIG) is derived from the blood of healthy blood donors and is made up of concentrated polyclonal immunoglobulin G (IgG). It was initially used as a treatment for patients with immunodeficiency. Nowadays, it is used as an off-label therapy for a wide variety of autoimmune and systemic inflammatory diseases, especially in dermatology. However, its mechanism of action is unknown [5-6]. Herein, we present a case of a female with multiple refractory LEP and DLE lesions over the face and scalp, respectively, which responded dramatically to IVIG.

## Case presentation

**Figure 1:**
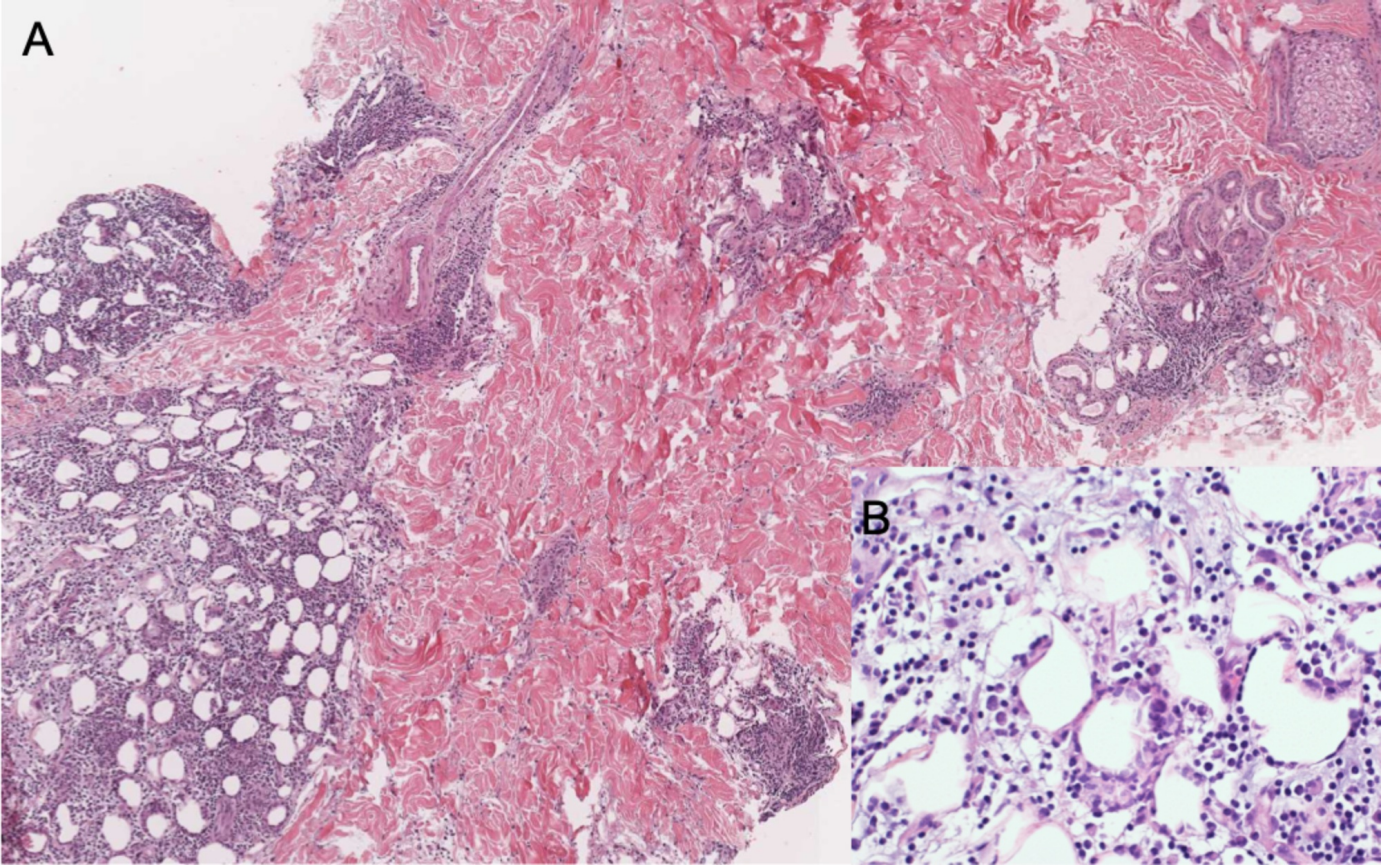
Thirty-six-year-old lady; Skin punch biopsy from the right cheek A: Section from subcutaneous tissue shows lobular panniculitis and perivascular and periadnexal lymphocytic infiltration. B: The infiltrate is predominantly lymphocytic with scattered plasma cells.

**Figure 2:**
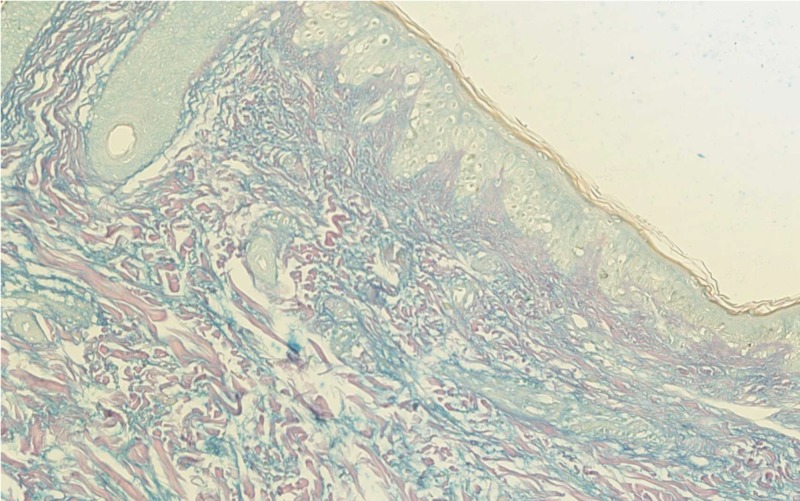
Thirty-six-year-old lady; Skin punch biopsy from the right cheek showing increased dermal mucin

In September 2016, a 34-year-old Saudi female was referred to the department of dermatology at King Faisal Specialist Hospital and Research Center (KFSHRC) with the aim of establishing a suspected diagnosis of LEP and for further management. The patient had been following up at multiple hospitals and has tried different treatments, including intralesional and systemic corticosteroids, hydroxychloroquine (HCQ), methotrexate, and mycophenolate mofetil with no satisfactory control over the lesions. On presentation, the main complaint of the patient was the presence of painful skin lesions over the face and two painful localized areas of alopecia over the scalp for more than 10 years. On physical examination, the patient had multiple, indurated, erythematous, tender plaques on the cheeks and one lesion on the forehead, measuring 0.5 cm in size. On her scalp, over the vertex, there were two well-defined erythematous, indurated, scarring patches of alopecia measuring 1.5 x 3 cm and 1 cm in size, with no other involved areas. Laboratory investigations conducted at the hospital revealed leucopenia, 3.89 x 109/L, and positive antinuclear antibodies (ANA) (titer 1:640 with a speckled pattern). However, anti-ds-DNA, anti-SSA (Ro), anti-Smith, anti-SCL-70, anti-JO1, and anti-RNP antibodies were negative. A skin biopsy taken from the cheek revealed lobular panniculitis with lymphocytic, including plasma cell infiltrates, dermal perivascular and periadnexal lymphocytic infiltrates with mucin deposition, follicular plugging, vacuolar-type interface dermatitis, and epidermal atrophy (Figure 1 and Figure 2).

**Figure 3:**
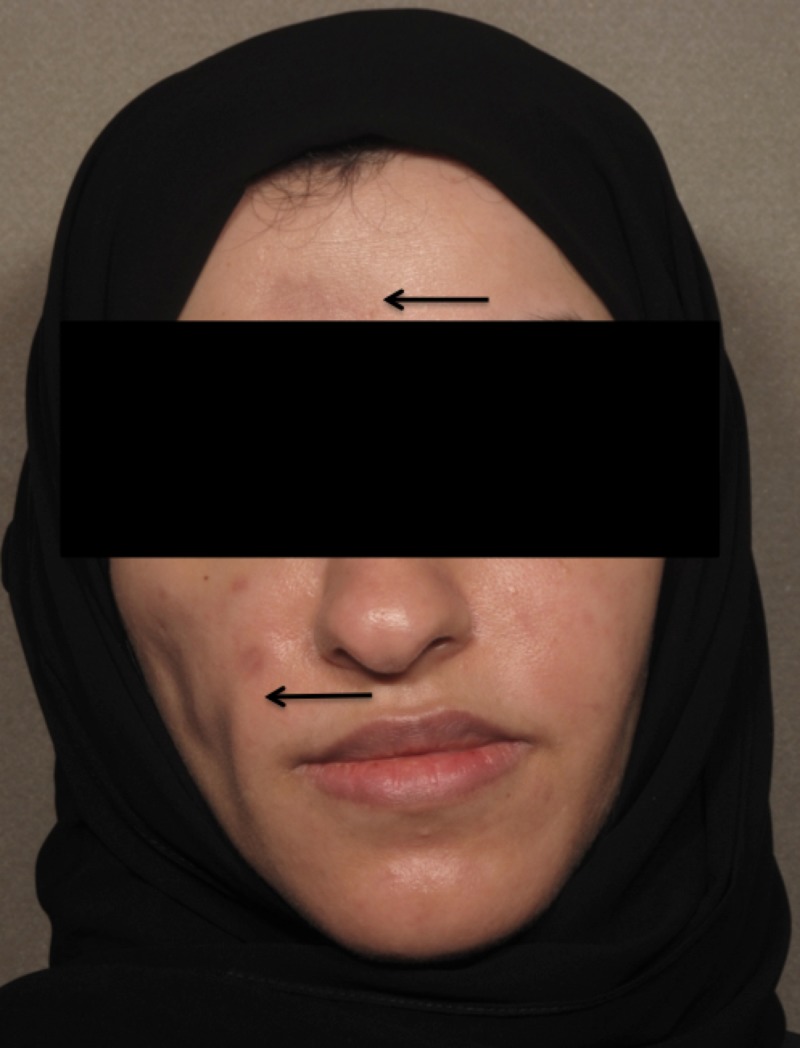
After four monthly cycles of intravenous immunoglobulin therapy Picture shows complete resolution of lupus panniculitis with residual lipoatrophy

**Figure 4:**
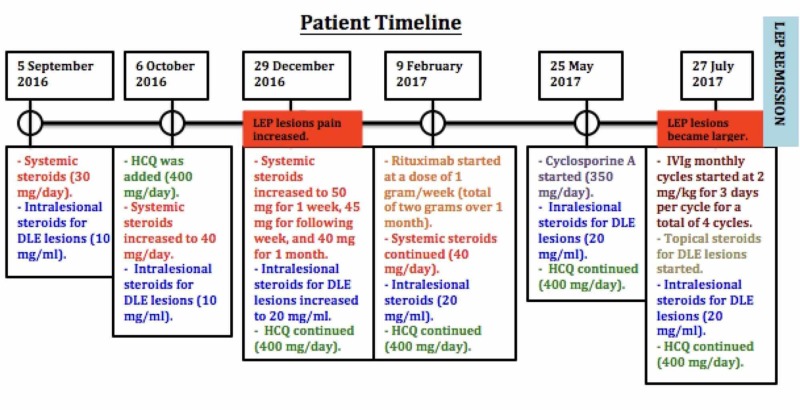
Patient treatment timeline

A biopsy from the scalp lesion revealed prominent fat necrosis with a membranocystic pattern, dermal periadnexal and perivascular lymphocytic infiltrate, follicular plugging, vacuolar-type interface dermatitis, and epidermal atrophy. The biopsy findings were consistent with the diagnosis of LEP over the cheeks and forehead and DLE over the scalp. During her follow-up, the patient was referred to the rheumatology clinic where she was cleared of SLE. Treatment was initiated with systemic corticosteroids at 30 mg/day, which was tapered down by 5 mg every three days, and intralesional corticosteroids 10 mg/ml for DLE lesions over the scalp. One month later, both LEP and DLE lesions were still painful and erythematous. Therefore, HCQ 400 mg/day was added and the systemic corticosteroids dose was increased to 40 mg/day, which was tapered down to 20 mg/day, and she was given intralesional corticosteroids for DLE lesions (10 mg/ml). Two months later, she was still complaining of pain and itching over the scalp lesions and pain over the facial lesions. The systemic corticosteroid dose was increased to 40 mg/day, which was tapered down to 30 mg/day. Intralesional corticosteroids and HCQ were continued. The patient was seen one month later, however, LEP lesions have become more painful and there was little improvement in the pain over the DLE lesions, so the systemic corticosteroids were increased to 50 mg/day for one week, followed by 45 mg/day for the next week, and 40 mg/day to be continued for two weeks. Also, the intralesional corticosteroids for DLE lesions were increased to 20 mg/ml and HCQ was continued. One month later, there was no improvement, thus, rituximab infusions at a rate of 1 gram every two weeks for a total dose of 2 grams were initiated. Systemic corticosteroids 40 mg/day, intralesional corticosteroids, and HCQ were continued. The patient was seen 15 weeks later, at which she had HCQ for seven months, systemic corticosteroids for nine months, more than four sessions of intralesional corticosteroids, and two rituximab infusions, the last being one month prior to her appointment; there was no improvement. Thus, cyclosporine 350-mg/day was started and monthly intralesional corticosteroids for DLE lesions and HCQ were continued. Two months later, she reported that she was unable to tolerate cyclosporine and discontinued its use after 20 days and that her facial lesions have grown larger. Therefore, IVIG monthly cycles were started three months after the last rituximab infusion at 2 mg/kg infused over three days per month along with topical and intralesional corticosteroids for DLE lesions and HCQ. After four cycles of IVIG, LEP lesions had resolved with residual hyperpigmentation and atrophy (Figure 3) and the DLE lesions over the scalp had some new hair regrowth (Figure 4). IVIG, topical and intralesional corticosteroids for DLE lesions, and HCQ were continued. After 12 cycles of IVIG, LEP lesions have remained in remission and no new lesions have developed. However, she was still complaining of pain and itching over the scalp lesions, which occur one week after receiving the intralesional corticosteroid injections, therefore, mycophenolate mofetil 1000 mg/day was added. It was later increased to 2000 mg/day due to the same complaint; also, intralesional corticosteroids were increased from 20 mg/ml to 40 mg/ml. IVIG tapering off will be initiated by increasing the intervals between single cycles by two weeks each time until a 16-week interval is reached where the drug will be discontinued; Moreover, HCQ, mycophenolate mofetil, and topical and intralesional corticosteroids for DLE lesions will be continued. Also, the patient was referred to plastic surgery for an auto-fat transplant.

## Discussion

LEP is a rare subtype of chronic CLE [7]. It was first described by Kaposi in 1883 and was later named “lupus erythematosus panniculitis” by Irgang in 1940 [8]. Although the terms LEP and lupus profundus are used interchangeably, it is suggested that only when the subcutaneous inflammatory process is accompanied by overlying DLE lesions is it called lupus profundus [7]. LEP most commonly affects women between the ages of 30 and 60 years. It is characterized by the presence of deep, tender subcutaneous nodules or plaques located in adipose-rich areas such as the arms and buttocks. Histopathologically, it is characterized by the presence of lymphocytic infiltrates and hyaline necrosis of the fat lobules [7-9]. The gold standard for establishing a diagnosis is by a histopathological examination of a lesional skin specimen. LEP has a relapsing course and new lesions may develop in previously affected areas or in new areas [10]. The healing of the lesions leaves behind irreversible lipoatrophy, which can be a major source of disfigurement and depression [11]. Commonly utilized treatments include antimalarial drugs, such as chloroquine or HCQ for mild cases of separate LEP. Topical and intralesional corticosteroids are also used. In refractory cases, methotrexate, cyclosporine A, cyclophosphamide, thalidomide, and the monoclonal antibody rituximab have been used [10-11]. IVIG is a fractionated blood product consisting of pooled IgG antibodies. Over 10,000 healthy blood donors are required for the production of a single batch. Initially, it was used for the treatment of primary and secondary immune deficiencies; however, its use has expanded tremendously in the past few decades. Effects produced by IVIG include the blockage and degradation of complement, induction of immunomodulatory Fc receptors, inhibition of B-cells, altering T-cell function, migration, and cytokine production. It is either used as a monotherapy or with an immunosuppressant [6]. There are multiple cases in the literature reporting the successful treatment of refractory DLE lesions using IVIG [6]. However, there is only one report in the literature reporting the successful treatment of LEP using IVIG [11]. The high cost of IVIG is a major drawback to its use [5]. The common side effects associated with IVIG administration include flu-like symptoms, such as flushing, nausea, fever, malaise, and lethargy; however, these side effects can be overcome easily by slowing down the infusion rate and by the administration of antihistamines and non-steroidal anti-inflammatory drugs (NSAIDs) [12]. In this case, the disease follows a chronic course and is resistant to multiple widely used treatments. Systemic corticosteroids, antimalarials, methotrexate, rituximab, cyclosporine A, and mycophenolate mofetil resulted in limited success. IVIG resulted in complete resolution of LEP lesions; however, DLE lesions required more aggressive immunosuppression using mycophenolate mofetil, which was added later.

## Conclusions

LEP is a rare variant of CLE. Antimalarial drugs, systemic corticosteroids, and immunosuppressive medications are often effective in the treatment of LEP. In this case, IVIG has proven effective when other modalities provided suboptimal control. Therefore, IVIG can be used when other standard therapy modalities have failed to control the disease. However, large clinical trials are needed to prove the efficacy of IVIG in the treatment of LEP.
